# Supplementary material for: Deep neural networks using a single neuron: folded-in-time architecture using feedback-modulated delay loops
Source: Nat Commun. 2021 Aug 27;12:5164. doi: 10.1038/s41467-021-25427-4 (PMC8397757; doi:10.1038/s41467-021-25427-4)
Supplement: Supplementary file 1 — Supplementary Information [file 41467_2021_25427_MOESM1_ESM.pdf]

Deep Neural Networks using a Single Neuron: Folded-in-Time Architecture using Feedback-Modulated Delay Loops

Supplementary Information

Florian Stelzer[1,2,3], André Röhm[4], Raul Vicente[3], Ingo Fischer[4], and Serhiy Yanchuk[1]

[1]Institute of Mathematics, Technische Universität Berlin, 10623, Germany
[2]Department of Mathematics, Humboldt-Universität zu Berlin, 12489, Germany
[3]Institute of Computer Science, University of Tartu, Estonia
[4]Instituto de Física Interdisciplinar y Sistemas Complejos, IFISC (UIB-CSIC), Campus Universitat de les Illes Baleares, E-07122 Palma de Mallorca, Spain

Supplementary Note 1: Fit-DNN performance and confusion matrices for different number of hidden layers, choice of delays, and activation functions

Supplementary Table 1 shows how the number of hidden layers L affects the performance of the Fit-DNN. We investigated two cases: the map limit $\theta \to \infty$ and the case $\theta = 0.5$. If the system operates in the map limit, we observe that the optimal number of hidden layers is 2 or 3, depending on the task. If $\theta = 0.5$, the performance of the Fit-DNN drops significantly for the CIFAR-10 [1], the coarse CIFAR-100 [1], the cropped SVHN [2], and the denoising task. For this reason, deeper networks do not offer an advantage for solving these tasks if $\theta = 0.5$. The MNIST [3] and Fashion-MNIST [4] accuracies do not suffer much from choosing a small node separation θ. Here the systems performance remains almost unchanged in comparison to the map limit.

Supplementary Figure 1 shows the effect of the choice of the number of delays D on the performance of the Fit-DNN. A larger number of delays D yields a slightly better accuracy for the CIFAR-10 task. We obtain an accuracy of less than 51% for $D = 25$, and an accuracy between 52% and 53% for $D = 125$ or larger. For the denoising task, we already obtain a good mean squared error (MSE) for a small number of delays D. The MSE remains mostly between 0.0253 and 0.0258 independently of D. The fluctuations of the MSE are small.

We compared two methods for choosing the delays $\tau_d = n_d\theta$. The first method is to draw the numbers n_d without replacement from a uniform distribution on the set $\{1, \ldots, 2N-1\}$. The second method is to choose equidistant delays, with $n_{d+1} - n_d = \lfloor (2N-1)/D \rfloor$. For the CIFAR-10 task, one may observe a slight advantage of the equidistant delays, whereas for the denoising task, randomly chosen delays yield slightly better results. In both cases, however, the influence of the chosen method on the quality of the results is small and seems to be insignificant.

Supplementary Table 2 compares the performance of the Fit-DNN for different activation functions $f(a) = \sin(a)$, $f(a) = \tanh(a)$, and $f(a) = \max(0, a)$ (ReLU). The results show that the Fit-DNN works well with various activation functions.

Supplementary Figure 2 shows the confusion matrices for the cropped SVHN and the CIFAR-10 tasks. These matrices show how often images of a corresponding dataset class are either recognized correctly or mismatched with another class. Confusion matrices are a suitable tool to identify which classes are confused more or less often. The confusion matrix for the cropped SVHN task shows, e.g., that the number 3 is relatively often falsely recognized as 5 or 9, but almost never as 4 or 6. The confusion matrix for the CIFAR-10 task indicates that images from animal classes (bird, cat, deer, dog, frog, horse) are often mismatched with another animal class, but rarely with a transportation class (airplane, automobile, ship, truck). This is an expected result for the CIFAR-10 task.

Supplementary Figure 3 shows results for a sine function fitting task. The objective of the task is to fit functions $y_i(u)$, $i = 1, \ldots, 5$, $u \in [-1, 1]$, plotted in Supplementary Figure 4, which are defined as concatenations $y_i(u) = s_i \circ \ldots \circ s_1(u)$ of sine functions $s_i(u) = \sin(\omega_i(u) + \varphi_i)$ with

$$\omega_1 = 0.65 \cdot 2\pi, \quad \omega_2 = 0.4 \cdot 2\pi, \quad \omega_3 = 0.3 \cdot 2\pi, \quad \omega_4 = 0.55 \cdot 2\pi, \quad \omega_5 = 0.45 \cdot 2\pi, \tag{1}$$

$$\varphi_1 = 1.0, \quad \varphi_2 = -0.5, \quad \varphi_3 = -0.3, \quad \varphi_4 = 0.6, \quad \varphi_5 = 0.2. \tag{2}$$

The simulations were performed with $N = 20$ nodes per hidden layer, $D = 3$, and $\tau_1 = 15$, $\tau_2 = 20$, $\tau_3 = 25$. Since the task is to fit a concatenation of i sine functions and the Fit-DNN consists in this case of L concatenated sine functions, one would expect optimal results for $L \geq i$. In our tests, this was true for up to $i = 3$ concatenated functions. The function y_1 can be approximated by the Fit-DNN's output with a small MSE with any number of layers, see Supplementary Figure 3. The function y_2 can be fitted with a small error if and only if $L \geq 2$ (with a few exceptions). For the function y_3 we obtain relatively exact approximations with 2 or more hidden layers, but the smallest MSE is obtained with $L = 3$ in most cases. The Fit-DNN fails to fit the functions y_4 and y_5 for all L.

	L	1	2	3	4	
$\theta = 0.5$	MNIST	98.43	98.54	98.3	98.24	[%]
	Fashion-MNIST	87.61	87.87	87.51	87.44	[%]
	CIFAR-10	52.35	52.13	52.05	51.32	[%]
	coarse CIFAR-100	33.52	33.22	32.51	31.32	[%]
	cropped SVHN	78.26	78.78	78.21	78.39	[%]
	denoising ($D = 5$)	0.0250	0.0254	0.0269	0.0362	[MSE]
	denoising ($D = 50$)	0.0251	0.0253	0.0269	0.0278	[MSE]
map limit	MNIST	98.41	98.62	98.47	98.58	[%]
	Fashion-MNIST	87.22	87.91	87.97	87.88	[%]
	CIFAR-10	53.69	54.57	54.28	54.15	[%]
	coarse CIFAR-100	35.13	35.69	35.77	36.48	[%]
	cropped SVHN	80.38	82.71	82.92	82.22	[%]
	denoising ($D = 5$)	0.0255	0.0244	0.0246	0.0250	[MSE]
	denoising ($D = 50$)	0.0257	0.0241	0.0243	0.0246	[MSE]

Supplementary Table 1: Accuracies [%] for the classification tasks and mean squared error for the denoising task for different numbers of hidden layers L. For a node separation of $\theta = 0.5$, two hidden layers seem to be optimal for the classification tasks (except CIFAR-10/100), and one hidden layer is sufficient for the denoising task. When the systems operates in the map limit $\theta \to \infty$, additional hidden layers can improve the performance.

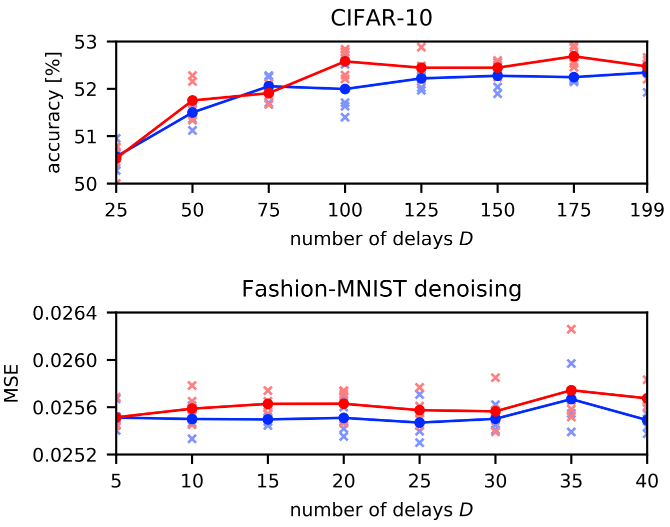

Supplementary Figure 1: Accuracy and MSE for different numbers of delays D. For each D, the plots show 5 results (cross symbols) with delays drawn from a uniform distribution (blue), and equidistant delays (red). The dot symbols connected by solid lines show the mean of the results.

f	sin	tanh	ReLU	
MNIST	98.396	98.611	98.833	[%]
Fashion-MNIST	87.921	88.78	89.177	[%]
CIFAR-10	52.062	51.039	50.598	[%]
coarse CIFAR-100	32.162	32.183	30.737	[%]
cropped SVHN	78.292	77.739	77.916	[%]
denoising	0.0254	0.0249	0.0255	[MSE]

Supplementary Table 2: Accuracies [%] for the classification tasks and mean squared error for the denoising task for different activation functions f. Overall, the compared activation functions work similarly well.

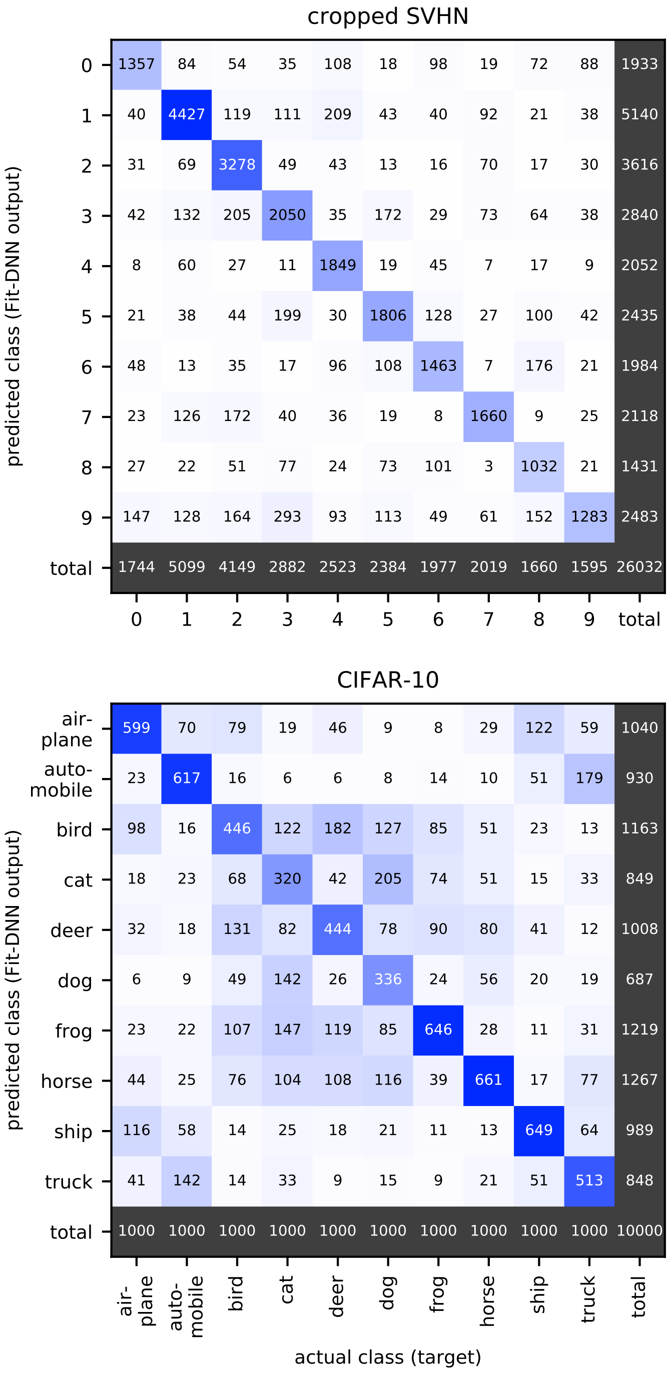

Supplementary Figure 2: Numbers of images from the cropped SVHN and CIFAR-10 test sets by their actual class and the Fit-DNN's prediction. The CIFAR-10 confusion matrix implies that false predictions occur mostly within the superclasses *animals* and *transportation* but rarely between the superclasses.

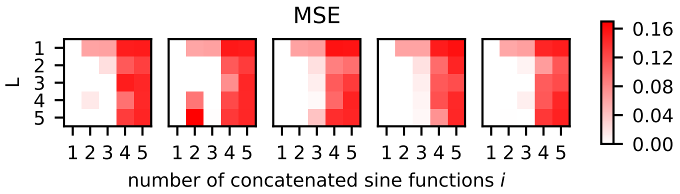

Supplementary Figure 3: The plot shows the mean squared errors (MSE) for fitting the functions $y_i(u)$, $i = 1, \dots, 5$ with different numbers of layers L. We repeated the numerical experiment five times, each panel shows the results of one of these independent repetitions.

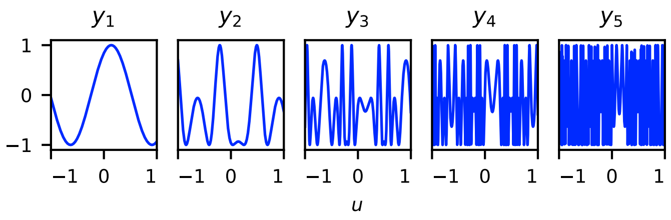

Supplementary Figure 4: Functions $y_i(u)$, $i = 1, \dots, 5$.

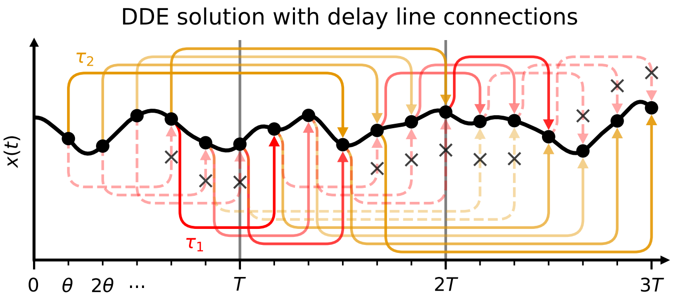

Supplementary Figure 5: Sketch of the solution of delay system (3)–(4) with delay-induced connections. Red arrows correspond to a delay $0 < \tau_1 < T$, and yellow to $T < \tau_2 < 2T$. Dashed lines with symbol $\times$ indicate connections that were removed by setting the modulation amplitude to zero; see Eq. (6).

Supplementary Note 2: The Fit-DNN delay system and network representation

2.1 Generating delay system

The Fit-DNN has M input nodes, P output nodes, and L hidden layers, each consisting of N nodes. The hidden layers are described by the delay system

$$\dot{x}(t) = -\alpha x(t) + f(a(t)), \tag{3}$$

$$a(t) = J(t) + b(t) + \sum_{d=1}^{D} \mathcal{M}_d(t) x(t - \tau_d), \tag{4}$$

where $\alpha > 0$ is a constant time-scale, f is a nonlinear activation function, and the argument $a(t)$ is a signal composed of a data signal $J(t)$, a bias signal $b(t)$, and delayed feedback terms modulated by functions $\mathcal{M}_d(t)$. The components of $a(t)$ are described in the Methods Section. The delays are given by $\tau_d = n_d\theta$, where $\theta := T/N$ and $1 \le n_1 < \ldots < n_D \le 2N-1$ are natural numbers. The state of the ℓ-th hidden layer is given by the solution $x(t)$ of (3)–(4) on the interval $(\ell-1)T < t \le \ell T$. We define the node states of the hidden layers as follows:

$$x_n^\ell := x((\ell-1)T + n\theta) \tag{5}$$

for the node $n = 1, \dots, N$ of the layer $\ell = 1, \dots, L$.

The nodes of the hidden layers are connected by the delays τ_d, as illustrated in Supplementary Figure 5. To ensure that only nodes of consecutive hidden layers are connected, we set

$$\mathcal{M}_d(t) = 0 \quad \text{if } t \in ((\ell-1)T, \ell T] \text{ and } t - \tau_d = t - n_d\theta \notin ((\ell-2)T, (\ell-1)T]. \tag{6}$$

The delay connections, which are set to zero by condition (6), are indicated by dashed arrows marked with a black $\times$ symbol in Supplementary Figure 5.

Additionally, we set $\mathcal{M}_d(t) = 0$ for $t \in [0, T]$. This implies, in combination with condition (6), that the system has no incoming delay connections from a time $t - \tau_d$ before zero. For this reason, a history function [5, 6, 7, 8] is not required to solve the delay system (3)–(4) for positive time. Knowing the initial condition $x(0) = x_0$ at a single point is sufficient.

System (3)–(4) is defined on the interval $[0, LT]$. The application of the variation of constants formula gives for $0 \le t_0 < t \le LT$ the equation

$$x(t) = e^{-\alpha(t-t_0)} x(t_0) + \int_{t_0}^{t} e^{\alpha(s-t)} f(a(s))\, \mathrm{d}s. \tag{7}$$

Using this equation on appropriate time intervals $[(n-1)\theta, n\theta]$, we obtain the following relations for the nodes in the first hidden layer

$$x_1^1 = e^{-\alpha\theta} x_0 + \int_0^\theta e^{\alpha(s-\theta)} f(a(s))\, \mathrm{d}s, \tag{8}$$

$$x_n^1 = e^{-\alpha\theta} x_{n-1}^1 + \int_0^\theta e^{\alpha(s-\theta)} f(a((n-1)\theta + s))\, \mathrm{d}s, \quad n = 2, \dots, N. \tag{9}$$

Here $x_0 = x(0)$ is the initial state of system (3)–(4). Similarly, for the hidden layers $\ell = 2, \dots, L$, we have

$$x_1^\ell = e^{-\alpha\theta} x_N^{\ell-1} + \int_0^\theta e^{\alpha(s-\theta)} f(a((\ell-1)T + s))\,\mathrm{d}s, \tag{10}$$

$$x_n^\ell = e^{-\alpha\theta} x_{n-1}^{\ell} + \int_0^\theta e^{\alpha(s-\theta)} f(a((\ell-1)T + (n-1)\theta + s))\,\mathrm{d}s, \quad n = 2, \dots, N. \tag{11}$$

For the first hidden layer, the signal $a(t)$ is piecewise constant. More specifically,

$$a(s) = J(s) = a_n^1 = g\left(a_n^{\mathrm{in}}\right), \quad (n-1)\theta < s \le n\theta,\ n = 1, \dots, N, \tag{12}$$

where

$$a_n^{\mathrm{in}} = w_{n,M+1}^{\mathrm{in}} + \sum_{m=1}^{M} w_{nm}^{\mathrm{in}} u_m. \tag{13}$$

Taking into account Eq. (12), relations (8)–(9) lead to the following exact expressions for the nodes of the first hidden layer:

$$x_1^1 = e^{-\alpha\theta} x_0 + \alpha^{-1}(1 - e^{-\alpha\theta}) f(a_1^1), \tag{14}$$

$$x_n^1 = e^{-\alpha\theta} x_{n-1}^1 + \alpha^{-1}(1 - e^{-\alpha\theta}) f(a_n^1), \quad n = 2, \dots, N. \tag{15}$$

2.2 Network representation for small node separations

For the hidden layers $\ell = 2, \dots, L$, i.e., for $T < t \le LT$, the signal $a(t)$ is defined by

$$a(t) = b(t) + \sum_{d=1}^{D} \mathcal{M}_d(t) x(t - \tau_d), \tag{16}$$

where $b(t)$ and $\mathcal{M}_d(t)$ are piecewise constant functions with discontinuities at the grid points $n\theta$. However, the feedback signals $x(t - \tau_d)$ are not piecewise constant. Therefore, we cannot replace $a((\ell-1)T + (n-1)\theta + s)$, $0 < s < \theta$, in Eq. (10) and (11) by constants. However, if the node separation θ is small, we can approximate the value of

$$x((\ell-1)T + (n-1)\theta + s - \tau_d) = x((\ell-1)T + (n - n_d - 1)\theta + s), \quad 0 < s < \theta, \tag{17}$$

by the value $x((\ell-1)T + n\theta - \tau_d)$, which can be rewritten as

$$x((\ell-1)T + n\theta - \tau_d) = x((\ell-1)T + (n - n_d)\theta) = x((\ell-2)T + (n - n'_d)\theta), \tag{18}$$

where $n'_d = n_d - N$. Condition (6) ensures that the layer $\ell - 1$ is connected only to the previous layer $\ell - 2$. Formally, it means the nonzero values $v_{d,n}^\ell$ of $\mathcal{M}_d$ allow only such connections that $1 \le n - n'_d \le N$, i.e., $x((\ell-2)T + (n - n'_d)\theta) = x_{n-n'_d}^{\ell-2}$. As a result, we can approximate $a((\ell-1)T + (n-1)\theta + s)$ for $0 < s < \theta$ by

$$a_n^\ell = w_{n,N+1}^\ell + \sum_{j=1}^{N} w_{nj}^\ell x_j^{\ell-1}, \quad n = 1, \dots, N,\ \ell = 2, \dots, L, \tag{19}$$

where

$$w_{nj}^\ell := \delta_{N+1,j} b_n^\ell + \sum_{d=1}^{D} \delta_{n-n'_d,j} v_{d,n}^\ell \tag{20}$$

defines a weight matrix $W^\ell = (w_{nj}^\ell) \in \mathbb{R}^{N \times (N+1)}$ for the connections from layer $\ell - 1$ to layer ℓ. This matrix is illustrated in Supplementary Figure 6.

In summary, we obtain the following network representation of the Fit-DNN, illustrated in Supplementary Figure 7, which approximates the node states up to first order terms in θ. The first hidden layer is given by

$$x_1^1 = e^{-\alpha\theta} x_0 + \alpha^{-1}(1 - e^{-\alpha\theta}) f(a_1^1), \tag{21}$$

$$x_n^1 = e^{-\alpha\theta} x_{n-1}^1 + \alpha^{-1}(1 - e^{-\alpha\theta}) f(a_n^1), \quad n = 2, \dots, N. \tag{22}$$

The hidden layers $\ell = 2, \dots, L$ are given by

$$x_1^\ell = e^{-\alpha\theta} x_N^{\ell-1} + \alpha^{-1}(1 - e^{-\alpha\theta}) f(a_1^\ell), \tag{23}$$

$$x_n^\ell = e^{-\alpha\theta} x_{n-1}^\ell + \alpha^{-1}(1 - e^{-\alpha\theta}) f(a_n^\ell), \quad n = 2, \dots, N, \tag{24}$$

and the output layer is defined by

$$\hat{y}_p := f_p^{\mathrm{out}}(\mathbf{a}^{\mathrm{out}}), \quad p = 1, \dots, P, \tag{25}$$

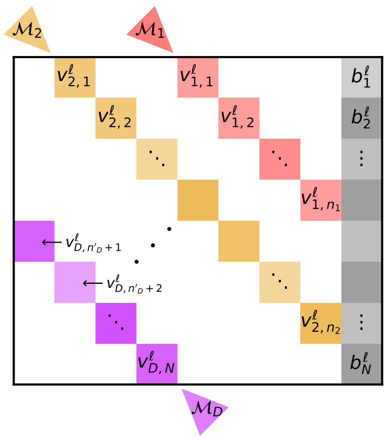

Supplementary Figure 6: Illustration of the sparse weight matrix W^ℓ containing the connection weights between the hidden layers $\ell - 1$ and ℓ, see Eq. (20). The nonzero weights are arranged on diagonals, and equal to the values $v^\ell_{d,n}$ of the functions $\mathcal{M}_d$. The position of the diagonals is determined by the corresponding delays τ_d. If $\tau_d = N\theta = T$, then the main diagonal contains the entries $v^\ell_{d,1}, \ldots, v^\ell_{d,N}$. If $\tau_d = n_d\theta < T$, then the corresponding diagonal lies above the main diagonal and contains the values $v^\ell_{d,1}, \ldots, v^\ell_{d,n_d}$. On the contrary, for $\tau_d = n_d\theta > T$, the corresponding diagonal lies below the main diagonal and contains the values $v^\ell_{d,n'_d+1}, \ldots, v^\ell_{d,N}$, where $n'_d = n_d - N$. The last column of the matrix contains the bias weights.

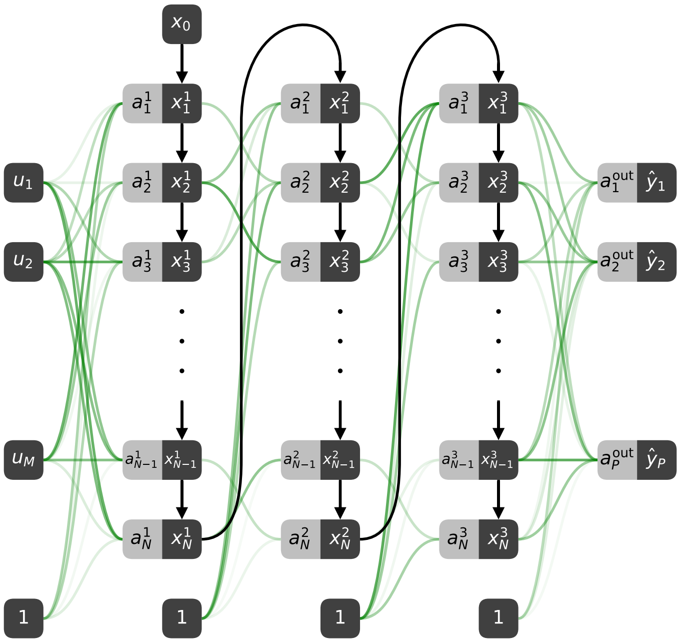

Supplementary Figure 7: The multilayer neural network described by the equations (21)–(28). Adaptable connection weights are plotted in green. The connections between the input layer and the first hidden layer as well as the connections between the last hidden and the output layer are dense (all-to-all connection). The hidden layers are in general sparsely connected; see Supplementary Figure 6 for an illustration of the connection matrices between the hidden layers. In contrast to a classical multilayer perceptron, the Fit-DNN comprises fixed linear connections between neighboring nodes (black arrows). These additional connections must be taken into account when computing the error gradients of the network. Note that the hidden layers, namely the nodes $x^{\ell-1}_N$ and x^ℓ_1, are also directly connected by such linear links.

where f^{out} is an output activation function which suits the given task. Moreover,

$$a_n^1 := g(a_n^{\text{in}}) := g\left(\sum_{m=1}^{M+1} w_{nm}^{\text{in}} u_m\right), \qquad n = 1, \ldots, N, \tag{26}$$

$$a_n^\ell := \sum_{j=1}^{N+1} w_{nj}^\ell x_j^{\ell-1}, \qquad n = 1, \ldots, N,\ \ell = 2, \ldots, L, \tag{27}$$

$$a_p^{\text{out}} := \sum_{n=1}^{N+1} w_{pn}^{\text{out}} x_n^L, \qquad p = 1, \ldots, P, \tag{28}$$

where $u_{M+1} := 1$ and $x_{N+1}^\ell := 1$, for $\ell = 1, \ldots, L$. We call the a_n^ℓ and a_p^{out} the activation of the corresponding node. For $n = 1, \ldots, N$, the variable w_{pn}^{out} denotes the output weight connecting the n-th node of layer L to the p-th output node, and $w_{p,(N+1)}^{\text{out}}$ denotes the bias for p-th output node (in other words, the weight connecting the on-neuron x_{N+1}^L of layer L to the p-th output node).

The topology of the obtained network representation of the Fit-DNN does not depend on the discretization method. Instead of the above derivation, one could simply approximate the node states by applying an Euler scheme to the delay system (3)–(4). The obtained map

$$x_n^\ell = x_{n-1}^\ell + \theta f(a_n^\ell), \tag{29}$$

possesses the same connections as the network representation (21)–(28) of the Fit-DNN, but has slightly different connection weights. Nevertheless, for our purposes it is necessary to consider (21)–(28) instead of the simple Euler scheme (29). The weights $e^{-\alpha\theta}$ of the linear connections of neighboring nodes in Eqs. (21)–(24) are only slightly smaller than the corresponding weights 1 in Eq. (29), but they allow to avoid destabilization during the computation of the error gradient of the Fit-DNN by back-propagation, and lead to accurate results.

2.3 Map limit

Here we show that the nodes of the Fit-DNN (8)–(11) can be approximated by the map limit

$$x_n^\ell = \alpha^{-1} f(a_n^\ell) \tag{30}$$

for large node separation θ, up to exponentially small terms $\mathcal{O}(e^{-\beta\theta})$ for all $0 < \beta < \alpha$. This limit corresponds to the approach for building networks of coupled maps from delay systems in [9, 10].

For the nodes of the first hidden layer, Eqs. (14)–(15) provide exact solutions for any θ. Hence, replacing θ by $r \in [0, \theta]$, we obtain for the values of $x(t)$ in the interval $[(n-1)\theta, n\theta]$

$$x((n-1)\theta + r) = e^{-\alpha r} x_{n-1}^1 + \alpha^{-1}(1 - e^{-\alpha r}) f(a_n^1), \tag{31}$$

which implies that the solution $x(t)$ decays exponentially to $\alpha^{-1} f(a_n^1)$. In other words, it holds

$$x((n-1)\theta + r) = \alpha^{-1} f(a_n^1) + \mathcal{O}(e^{-\alpha r}). \tag{32}$$

To show similar exponential estimates for the layers $\ell = 2, \ldots, L$, we use inductive arguments. For this, we assume that the following estimate holds for layer $\ell - 1$:

$$x((\ell-2)T + (n-1)\theta + r) = \alpha^{-1} f(a_n^{\ell-1}) + \mathcal{O}(e^{-\beta r}) \tag{33}$$

for all $0 < \beta < \alpha$, $r \in [0, \theta]$, and all n within the layer. Note that this estimate is true for the first hidden layer because (33) is a weaker statement than (32). For layer ℓ, we obtain from Eq. (7)

$$x((\ell-1)T + (n-1)\theta + r) = e^{-\alpha r} x_{n-1}^\ell + \int_0^r e^{\alpha(s-r)} f(a((\ell-1)T + (n-1)\theta + s))\, \mathrm{d}s, \tag{34}$$

where (33) implies

$$\begin{aligned} a((\ell-1)T + (n-1)\theta + s) &= b((\ell-1)T + (n-1)\theta + s) + \sum_{d=1}^{D} \mathcal{M}_d^\ell((\ell-1)T + (n-1)\theta + s) x((\ell-1)T + (n-1)\theta + s - \tau_d) \\ &= b_n^\ell + \sum_{d=1}^{D} v_{d,n}^\ell x((\ell-2)T + (n-1)\theta - n_d'\theta + s) \\ &= b_n^\ell + \sum_{d=1}^{D} v_{d,n}^\ell x((\ell-2)T + (n - n_d')\theta) + \mathcal{O}(e^{-\beta s}) \\ &= a_n^\ell + \mathcal{O}(e^{-\beta s}). \end{aligned} \tag{35}$$

We obtain the term $\mathcal{O}(e^{-\beta s})$ in Eq. (35) because Eq. (33) implies

$$x((\ell-2)T + (n-1)\theta - n_d'\theta + s) + \mathcal{O}(e^{-\beta s}) = \alpha^{-1} f(a_{n-n_d'}^{\ell-1}) = x((\ell-2)T - (n - n_d')\theta) + \mathcal{O}(e^{-\beta\theta}) \tag{36}$$

and $e^{-\beta\theta} < e^{-\beta s}$. If f is Lipschitz continuous (which is the case for all our examples), it follows from Eqs. (34) and (35) that

$$x((\ell-1)T + (n-1)\theta + r) = e^{-\alpha r} x_{n-1}^{\ell} + \alpha^{-1}(1 - e^{-\alpha r}) f(a_n^{\ell}) + \int_0^r e^{\alpha(s-r)} \mathcal{O}(e^{-\beta s})\, \mathrm{d}s. \tag{37}$$

Since

$$\int_0^r e^{\alpha(s-r)} e^{-\beta s}\, \mathrm{d}s = \frac{1}{\alpha-\beta}(e^{-\beta r} - e^{-\alpha r}) < \frac{e^{-\beta r}}{\alpha - \beta}, \tag{38}$$

we obtain

$$x((\ell-1)T + (n-1)\theta + r) = \alpha^{-1} f(a_n^{\ell}) + \mathcal{O}(e^{-\beta r}). \tag{39}$$

This holds in particular for $r = \theta$. Therefore, we have shown that Eq. (30) holds up to terms of order $\mathcal{O}(e^{-\beta\theta})$ for all $0 < \beta < \alpha$.

Supplementary Note 3: Back-propagation for the Fit-DNN

To calculate the error gradient of a traditional multilayer perceptron, it sufficient to compute partial derivatives of the loss function with respect to the node activations $\partial\mathcal{E}/\partial a_n^{\ell}$ by an iterative application of the chain rule and to store them as intermediate results. These derivatives are called *error signals* and are denoted by δ_n^{ℓ}. Subsequently, the weight gradient can be calculated by applying the chain rule again for each weight, i.e., the *back-propagation*.

The network representation (21)–(28) of the Fit-DNN, illustrated by Supplementary Figure 7, contains additional linear connections which are not present in classical multilayer perceptrons. We need to take these connections into account when calculating the weight gradient of the loss function, more specifically, the error signals δ_n^{ℓ}. Despite having these additional connections, all nodes are still strictly forward-connected. Consequently, we can calculate the error signals by applying the chain rule node by node. Thereby, we employ a second type of error signal $\Delta_n^{\ell} := \partial\mathcal{E}/\partial x_n^{\ell}$ because the local connections (black arrows in Supplementary Figure 7) do not enter the nodes through the activation function. Thus, we need to know Δ_n^{ℓ} for the back-propagation via these local connections. However, memory efficient implementations are possible because we only need to store one Δ_n^{ℓ} at a time. The weight gradient can again be calculated from the error signals δ_n^{ℓ} by using the chain rule once more for each weight.

The back-propagation algorithm for the Fit-DNN is described in the Methods Section. In the following we explain the Steps 1–4 of this algorithm in detail.

Step 1: For certain favorable choices of the loss function $\mathcal{E}$ and the output activation function f^{out}, we can compute the error signal of the output layer by the following simple equation:

$$\delta_p^{\text{out}} = \frac{\partial\mathcal{E}}{\partial a_p^{\text{out}}} = \hat{y}_p - y_p, \tag{40}$$

for $p = 1, \ldots, P$. This holds in particular for combining the cross-entropy loss function with the softmax output function and for combining the mean-squared loss function with the identity output function. For a derivation we refer to [11] or [12].

Step 2: The formulas for the error signals of the last hidden layer can be found by applying the chain rule twice. Let $\Phi := \alpha^{-1}(1 - e^{-\alpha\theta})$. The error derivatives w.r.t. the node states of the last hidden layer can be calculated from the output error signals and the output weight. We have

$$\Delta_N^L = \frac{\partial\mathcal{E}}{\partial x_N^L} = \sum_{p=1}^{P} \frac{\partial\mathcal{E}}{\partial a_p^{\text{out}}} \frac{\partial a_p^{\text{out}}}{\partial x_N^L} = \sum_{p=1}^{P} \delta_p^{\text{out}} w_{pN}^{\text{out}}, \tag{41}$$

and

$$\Delta_n^L = \frac{\partial\mathcal{E}}{\partial x_n^L} = \frac{\partial\mathcal{E}}{\partial x_{n+1}^L}\frac{\partial x_{n+1}^L}{\partial x_n^L} + \sum_{p=1}^{P} \frac{\partial\mathcal{E}}{\partial a_p^{\text{out}}} \frac{\partial a_p^{\text{out}}}{\partial x_n^L} = \Delta_{n+1}^L e^{-\alpha\theta} + \sum_{p=1}^{P} \delta_p^{\text{out}} w_{pn}^{\text{out}}, \tag{42}$$

for $n = N-1, \ldots, 1$. The error derivatives w.r.t. the node activations can then be calculated by multiplication with the corresponding derivative of the activation function, i.e.,

$$\delta_n^L = \frac{\partial\mathcal{E}}{\partial a_n^L} = \frac{\partial\mathcal{E}}{\partial x_n^L}\frac{\partial x_n^L}{\partial a_n^L} = \Delta_n^L \Phi f'(a_n^L), \tag{43}$$

for $n = 1, \ldots, N$.

Step 3: Also for the remaining hidden layers, we need only to apply the chain rule twice to obtain the formulas for the error signals. For $\ell = L-1, \ldots, 1$, we have

$$\Delta_N^{\ell} = \frac{\partial\mathcal{E}}{\partial x_N^{\ell}} = \frac{\partial\mathcal{E}}{\partial x_1^{\ell+1}}\frac{\partial x_1^{\ell+1}}{\partial x_N^{\ell}} + \sum_{i=1}^{N} \frac{\partial\mathcal{E}}{\partial a_i^{\ell+1}} \frac{\partial a_i^{\ell+1}}{\partial x_N^{\ell}} = \Delta_1^{\ell+1} e^{-\alpha\theta} + \sum_{i=1}^{N} \delta_i^{\ell+1} w_{iN}^{\ell+1}, \tag{44}$$

and

$$\Delta_n^{\ell} = \frac{\partial\mathcal{E}}{\partial x_n^{\ell}} = \frac{\partial\mathcal{E}}{\partial x_{n+1}^{\ell}}\frac{\partial x_{n+1}^{\ell}}{\partial x_n^{\ell}} + \sum_{i=1}^{N} \frac{\partial\mathcal{E}}{\partial a_i^{\ell+1}} \frac{\partial a_i^{\ell+1}}{\partial x_n^{\ell}} = \Delta_{n+1}^{\ell} e^{-\alpha\theta} + \sum_{i=1}^{N} \delta_i^{\ell+1} w_{in}^{\ell+1}, \tag{45}$$

for $n = N-1, \dots, 1$. Again, the error derivatives w.r.t. the node activations can be calculated by multiplication with the derivative of the activation function:

$$\delta_n^\ell = \frac{\partial \mathcal{E}}{\partial a_n^\ell} = \frac{\partial \mathcal{E}}{\partial x_n^\ell}\frac{\partial x_n^\ell}{\partial a_n^\ell} = \Delta_n^\ell \Phi f'(a_n^\ell), \tag{46}$$

for $n = 1, \dots, N$.

Step 4: Knowing the error signals, we can compute the weight gradient, i.e., the partial derivatives of the loss function w.r.t. the training parameters. For the partial derivatives w.r.t. the output weights, we obtain

$$\frac{\partial \mathcal{E}(\mathcal{W})}{\partial w_{pn}^{\text{out}}} = \frac{\partial \mathcal{E}}{\partial a_p^{\text{out}}}\frac{\partial a_p^{\text{out}}}{\partial w_{pn}^{\text{out}}} = \delta_p^{\text{out}} x_n^L, \tag{47}$$

for $n = 1, \dots, N+1,\ p = 1, \dots, P$. For the partial derivatives w.r.t. the hidden weights, it holds

$$\frac{\partial \mathcal{E}(\mathcal{W})}{\partial w_{nj}^\ell} = \frac{\partial \mathcal{E}}{\partial a_n^\ell}\frac{\partial a_n^\ell}{\partial w_{nj}^\ell} = \delta_n^\ell x_j^{\ell-1}, \tag{48}$$

for $j = 1, \dots, N+1,\ n = 1, \dots, N$. For the partial derivatives w.r.t. the input weights, the chain rule implies

$$\frac{\partial \mathcal{E}(\mathcal{W})}{\partial w_{nm}^{\text{in}}} = \frac{\partial \mathcal{E}}{\partial a_n^1}\frac{\partial a_n^1}{\partial w_{nm}^{\text{in}}} = \delta_n^1 \frac{\partial a_n^1}{\partial a_n^{\text{in}}}\frac{\partial a_n^{\text{in}}}{\partial w_{nm}^{\text{in}}} = \delta_n^1 g'(a_n^{\text{in}}) u_m, \tag{49}$$

for $m = 1, \dots, M+1,\ n = 1, \dots, N$.

The sums in Eq. (44) and Eq. (45) can be rewritten as sums over the index d of the delays:

$$\sum_{i=1}^{N} \delta_i^{\ell+1} w_{in}^{\ell+1} = \sum_{\substack{d=1 \\ 1 \le n+n'_d \le N}}^{D} \delta_{n+n'_d}^{\ell+1} v_{d,n+n'_d}^{\ell+1}. \tag{50}$$

This way we achieve a substantially faster computation if the number of delays D is much smaller than the number of nodes per hidden layer N. Equation (50) is obtained by exploiting the special sparsity structure of the weight matrices W^ℓ, $\ell = 2, \dots, L$. The entries of these matrices are defined by Eq. (20), which we rewrite here using the indices of $w_{in}^{\ell+1}$ from Eq. (50):

$$w_{in}^{\ell+1} = \delta_{N+1,n} b_i^{\ell+1} + \sum_{d=1}^{D} \delta_{i-n'_d,n} v_{d,n+n'_d}^{\ell+1}. \tag{51}$$

Since we have $1 \le n \le N$ in Eq. (44) and Eq. (45), the weight $w_{in}^{\ell+1}$ is non-zero only if there is an index $d \in 1, \dots, D$ such that $i - n'_d = n$, or equivalently $i = n + n'_d$. In this case we have $w_{in}^{\ell+1} = v_{d,i}^{\ell+1} = v_{d,n+n'_d}^{\ell+1}$. On the contrary, for any index d, the value $v_{d,n+n'_d}^{\ell+1}$ defines a matrix element of $W^{\ell+1}$ if and only if $1 \le n + n'_d \le N$. This implies Eq. (50).

Supplementary Note 4: Time signals of the Fit-DNN

Supplementary Figure 8 illustrates how the Fit-DNN processes information by showing its time signals. Panel (a) illustrates the process of obtaining the data signal $J(t)$ from an input image from the MNIST dataset, in this case an image of the handwritten number 4. $J(t)$ is a step function with step size θ. First, the extended input vector $\mathbf{u}$ is multiplied by the trained input matrix W^{in}. Then an input preprocessing function g is applied element-wise to the entries of the obtained vector. The resulting values are the step heights of the data signal $J(t)$.

Panel (b) shows the internal processes in the hidden layers. From top to bottom we plot:

- the state of the system $x(t)$,
- the signal $a(t)$,
- the signal $a(t)$ decomposed into its components (i.e., the data signal, the modulated feedback signals, and the bias signal) indicated by their corresponding color,
- the data signal $J(t)$,
- the delayed feedback signals $x(t - \tau_d)$ (grey),
- the trained modulation functions $\mathcal{M}_d(t)$ (colored),
- and the bias $b(t)$.

The signal $a(t)$ for the first hidden layer, $0 \le t \le T$, coincides with the data signal $J(t)$. For the remaining hidden layers, the signal $a(t)$ is a sum of the modulated feedback signals and the bias.

Panel (c) illustrates the output layer. The vector $\mathbf{x}_L$, containing the values of $x(t)$ sampled at $t = (L-1)T+\theta, \dots, (L-1)T+N\theta$, is multiplied by the trained output matrix W^{out} to obtain the output activation vector. Then the softmax function is applied to obtain the output vector $\mathbf{y}^{\text{out}}$. In this case, the Fit-DNN correctly identifies the input as an image showing the number 4.

The training process, which leads to the trained system depicted in Supplementary Figure 8, is shown in Supplementary Movie 1.

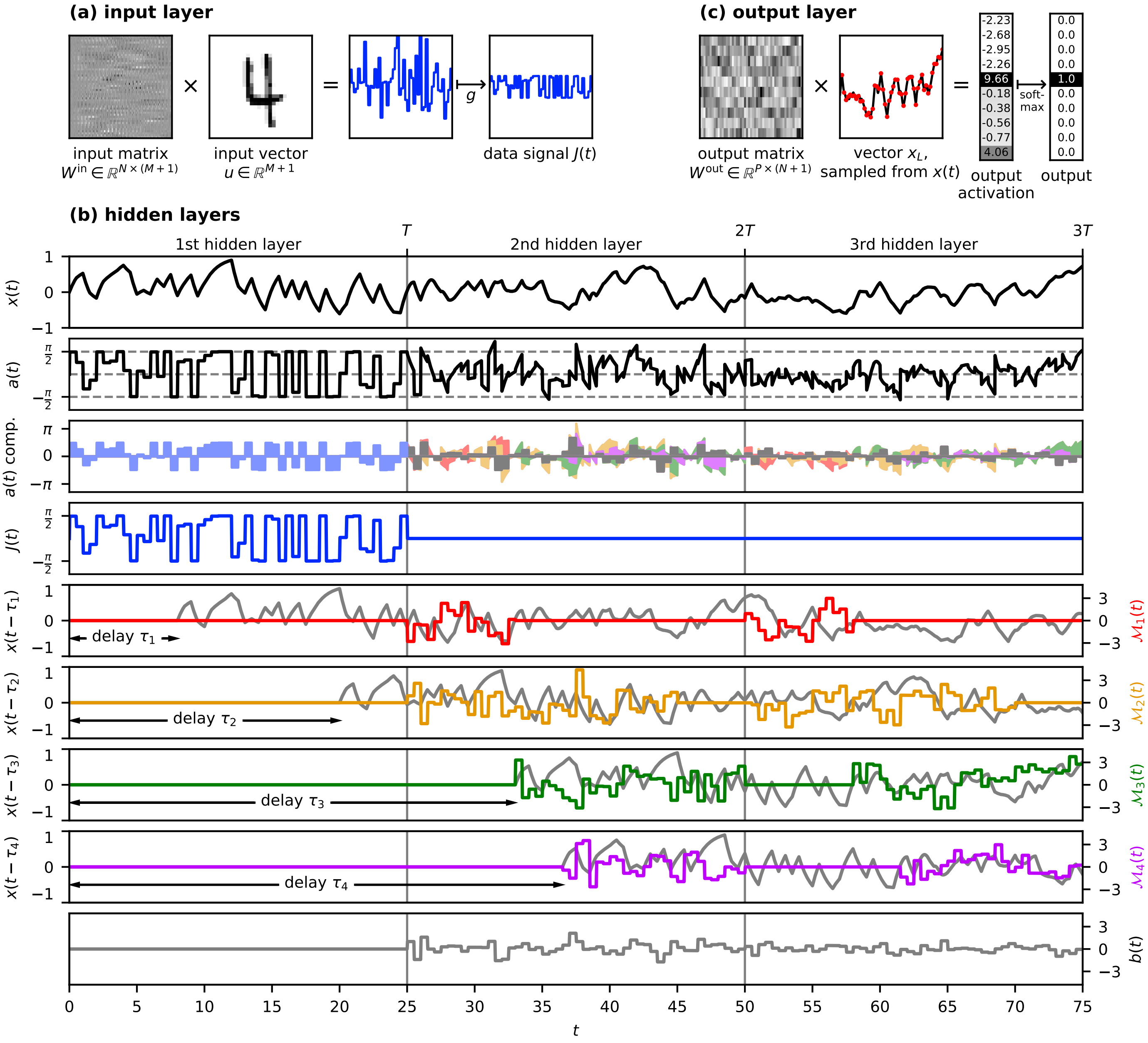

Supplementary Figure 8: Time signals of the Fit-DNN after training. Panel (a) illustrates the processing of an input image to obtain the data signal $J(t)$. Panel (b) shows the internal processes of the hidden layers: the state variable of the system $x(t)$, and the signal $a(t)$, which consists of the data signal $J(t)$, the delayed feedback signals $x(t-\tau_d)$ multiplied by the modulation functions $\mathcal{M}_d(t)$, and the bias signal $b(t)$. Panel (c) illustrates the output layer.

Supplementary References

[1] Krizhevsky, A. Learning multiple layers of features from tiny images. *University of Toronto* (2012).

[2] Netzer, Y. *et al.* Reading digits in natural images with unsupervised feature learning. *NIPS* (2011).

[3] Lecun, Y., Bottou, L., Bengio, Y. & Haffner, P. Gradient-based learning applied to document recognition. *Proceedings of the IEEE* **86**, 2278–2324 (1998).

[4] Xiao, H., Rasul, K. & Vollgraf, R. Fashion-mnist: a novel image dataset for benchmarking machine learning algorithms. Preprint at https://arxiv.org/abs/1708.07747 (2017).

[5] Hale, J. K. & Lunel, S. M. V. *Introduction to Functional Differential Equations* (Springer, New York, 1993).

[6] Diekmann, O., Verduyn Lunel, S. M., van Gils, S. A. & Walther, H.-O. *Delay Equations* (Springer, New York, 1995).

[7] Wu, J. *Introduction to Neural Dynamics and Signal Transmission Delay* (Walter de Gruyter, Berlin, Boston, 2001).

[8] Erneux, T., Javaloyes, J., Wolfrum, M. & Yanchuk, S. Introduction to Focus Issue: Time-delay dynamics. *Chaos: An Interdisciplinary Journal of Nonlinear Science* **27**, 114201 (2017).

[9] Hart, J. D., Schmadel, D. C., Murphy, T. E. & Roy, R. Experiments with arbitrary networks in time-multiplexed delay systems. *Chaos: An Interdisciplinary Journal of Nonlinear Science* **27**, 121103 (2017).

[10] Hart, J. D., Larger, L., Murphy, T. E. & Roy, R. Delayed dynamical systems: networks, chimeras and reservoir computing. *Philosophical Transactions of the Royal Society A: Mathematical, Physical and Engineering Sciences* **377**, 20180123 (2019).

[11] Bishop, C. M. *Pattern Recognition and Machine Learning* (Springer, New York, 2006).

[12] Goodfellow, I., Bengio, Y. & Courville, A. *Deep Learning* (MIT Press, Cambridge, Massachusetts, London, England, 2016).
