# Supplementary material for: Deep neural networks using a single neuron: folded-in-time architecture using feedback-modulated delay loops
Source: Nat Commun. 2021 Aug 27;12:5164. doi: 10.1038/s41467-021-25427-4 (PMC8397757; doi:10.1038/s41467-021-25427-4)
Supplement: Supplementary file 3 — Description of Additional Supplementary Files [file 41467_2021_25427_MOESM3_ESM.docx]

Description of Additional Supplementary Files

Title: Supplementary Movie 1

Description: Illustrates the training process, which leads to the trained system depicted in Supplementary Figure 8.

Panel (a) illustrates the process of obtaining the data signal 𝐽(𝑡) from an input image from the MNIST dataset, in this case an image of the handwritten number 4. 𝐽(𝑡) is a step function with step length θ. First, the extended input vector 𝑢 is multiplied by an input matrix, which changes during the training process. Then an input preprocessing function 𝑔is applied element-wise to the entries of the obtained vector. The resulting values are the step heights of the data signal 𝐽(𝑡).

Panel (b) shows the internal processes in the hidden layers. From top to bottom we plot:

• the state of the system 𝑥(𝑡),

• the signal 𝑎(𝑡),

• the signal 𝑎(𝑡) decomposed into its components (i.e., the data signal, the modulated feedback signals, and the bias signal) indicated by their corresponding color,

• the data signal 𝐽(𝑡),

• the delayed feedback signals 𝑥(𝑡 − τ (grey), 𝑑 )

• the modulation functions ℳ (colored), which 𝑑 (𝑡) are subject to training,

• and the bias 𝑏(𝑡). The signal a(t) for the first hidden layer, 0 < 𝑡 < 𝑇, coincides with the data signal 𝐽(𝑡). For the remaining hidden layers, the signal 𝑎(𝑡) is a sum of the modulated feedback signals and the bias.

Panel (c) illustrates the output layer. The vector 𝑥 , 𝐿 containing the values of 𝑥(𝑡) sampled from the last hidden layer, is multiplied by the output matrix 𝑊 𝑜𝑢𝑡 to obtain the output activation vector. The output matrix is subject to training, hence, the output weights change during the training process. Finally, the softmax function is applied to obtain the output vector 𝑦 . When the training process is finished, 𝑜𝑢𝑡 the Fit-DNN correctly identifies the input as an image showing the number 4.
